# Emergency Resection of a Bleeding Ruptured Malignant Phyllodes Tumor of the Breast

**DOI:** 10.7759/cureus.52775

**Published:** 2024-01-23

**Authors:** Hirokazu Matsushima, Masayuki Kikuchi, Rika Miyabe, Koji Atsuta, Tsunehiro Shintani

**Affiliations:** 1 Surgery, Japanese Red Cross Shizuoka Hospital, Shizuoka, JPN; 2 Surgery, Tosen Clinic, Shizuoka, JPN

**Keywords:** ruptured tumor, breast mass, emergency surgery, active bleeding, phyllodes tumor

## Abstract

Ruptured phyllodes tumors, though extremely rare, can necessitate emergency surgery in certain cases, particularly those with active bleeding. A 51-year-old woman presented to our hospital with a newly identified mass in her right breast that developed over the past two months. The tumor had ruptured through the paramammary nipple. While initially diagnosed with a phyllodes tumor and scheduled for elective surgery, she experienced active bleeding from the ruptured tumor, leading to a drop in hemoglobin levels. An emergency right simple mastectomy was performed to control the bleeding. Postoperatively, no complications or recurrences were observed. Phyllodes tumors, which are characterized by rapid growth, may present with active bleeding following rupture and may require emergency surgery.

## Introduction

Phyllodes tumors are rare, accounting for less than 0.3-0.9% of breast cancers [1]. They are classified as benign, borderline, or malignant, with malignant phyllodes tumors accounting for 25% of the total [2]. Phyllodes tumors occur in individuals with a wide age range, with the average age of diagnosis reported as 40-50 years [3-4]. These tumors have a high postoperative local recurrence rate, which is comparable among the three subtypes (median recurrence: 14.7-22.5 months for benign, 16.9-21 months for borderline, and 12-25 months for malignant), thus necessitating regular follow-up for two to three years after surgical resection [4]. Preoperative diagnosis is difficult as phyllodes tumors have a well-defined border appearance and resemble benign tumors, including fibroadenomas. Therefore, a phyllodes tumor should be suspected if the mass is greater than 3 cm or grows rapidly [5]. Although a needle biopsy may not achieve a definitive diagnosis [4], the fibromyxoid stromal fragments with spindle nuclei, fibroblastic pavements, and fibroblastic spindle cells on histopathological examination increase the diagnostic yield [5].

The standard treatment for a phyllodes tumor is surgical excision with a clear margin of healthy tissue (usually 1 cm) around it, which is associated with a low local recurrence rate [6]. Hematogenous metastasis is common for phyllodes tumors, whereas lymph node metastasis is rare; among cases wherein axillary surgery was performed, the positive rate was 0.1% after breast-conserving surgery and 2.1% after mastectomy [7]. Therefore, routine axillary lymph node dissection is considered unnecessary [4]. Moreover, adequate evidence for additional postoperative chemotherapy or radiation therapy is lacking [4].

Phyllodes tumors are characterized by rapid growth and, in rare cases, tumor rupture. Here, we present an extremely rare case of a ruptured malignant phyllodes tumor with active bleeding, which was successfully managed with an emergency simple mastectomy to achieve hemostasis.

## Case presentation

**Figure 1:**
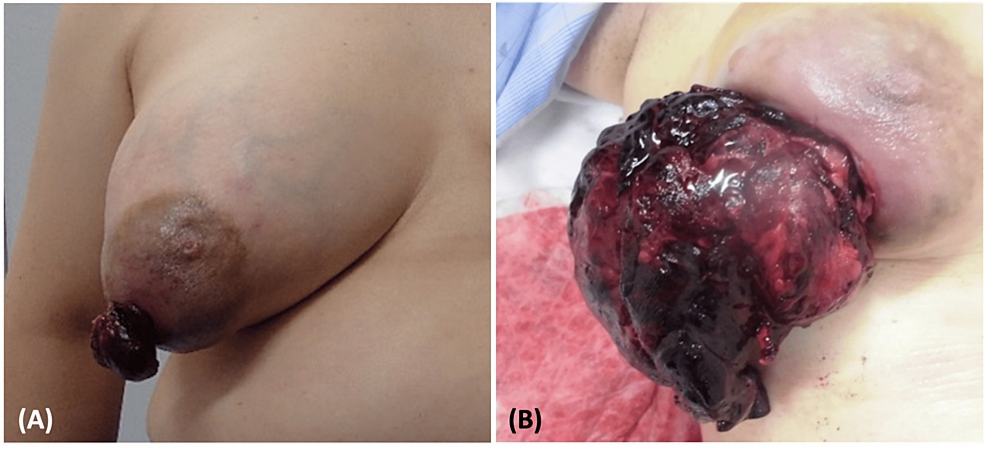
Appearance of ruptured phyllodes tumor (A) A right breast mass erupting from the paramammary nipple. (B) Three days later, increasing erupted mass and active bleeding from the erupting mass developed.

**Figure 2:**
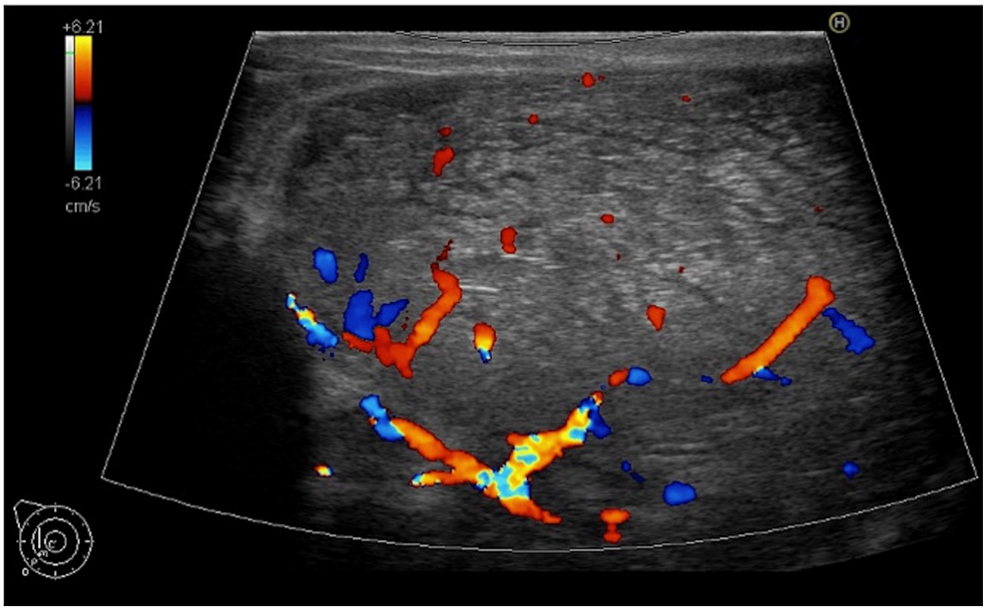
Breast ultrasonography and histopathological examination Breast ultrasonography showed a lobulated mass with well-defined borders and abundant internal blood flow in the right breast.

**Figure 3:**
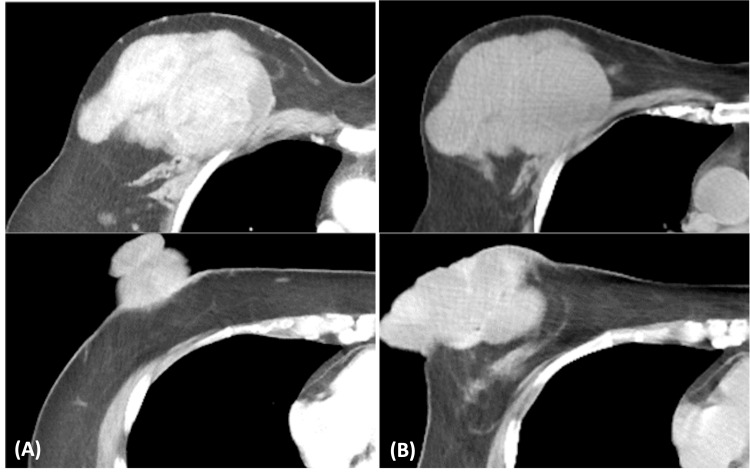
Computed tomography of the ruptured phyllodes tumor (A) Contrast-enhanced computed tomography (CT) findings at the initial examination findings demonstrated a 110×85 mm mass with contrast effects and calcification compressing the skin and pectoralis major muscle and a 40×35 mm mass erupting from the paramammary nipple. (B) CT findings three days after the initial examination showed a 105×75 mm mass with contrast effects and a 100×45 mm mass erupting from the paramammary nipple.

A 51-year-old woman with no significant medical history had noticed a mass in her right breast accompanied by breast tension two months before visiting our hospital but had neglected it. Thereafter, the mass in her right breast rapidly enlarged over the next two months, erupting from the paramammary nipple, prompting her to visit our hospital (Figure 1A). The patient did not complain of breast pain. Upon initial examination, the patient's right breast was entirely occupied by a fist-sized mass. The mass was described as elastic and mobile, with a 30×30 mm portion erupting from the paramammary nipple. Oozing was evident from the ruptured area, with a small amount of discharge adhering to her clothing. Blood test results revealed a hemoglobin (Hb) level of 13.2 g/dL and did not show elevation of tumor markers such as carcinoembryonic antigen (CEA) or carbohydrate antigen (CA) 15-3. Breast ultrasonography findings revealed a 90×80 mm lobulated mass with well-defined borders and abundant internal blood flow in the right breast (Figure 2). Contrast-enhanced computed tomography (CT) findings revealed a 110×85 mm mass with contrast effects and calcification compressing the skin and pectoralis major muscle and a 40×35 mm mass erupting from the paramammary nipple without extravasation of contrast indicating bleeding (Figure 3A). There was no evidence of distant metastasis or enlarged axillary lymph nodes.

**Table 1:** Blood test

Blood test	Result	Reference range
Hemoglobin	13.2 g/dL	12-16 g/dL
CEA	1.75 ng/mL	0.1-5.0 ng/mL
CA15-3	13 U/mL	<27 U/mL

**Figure 4:**
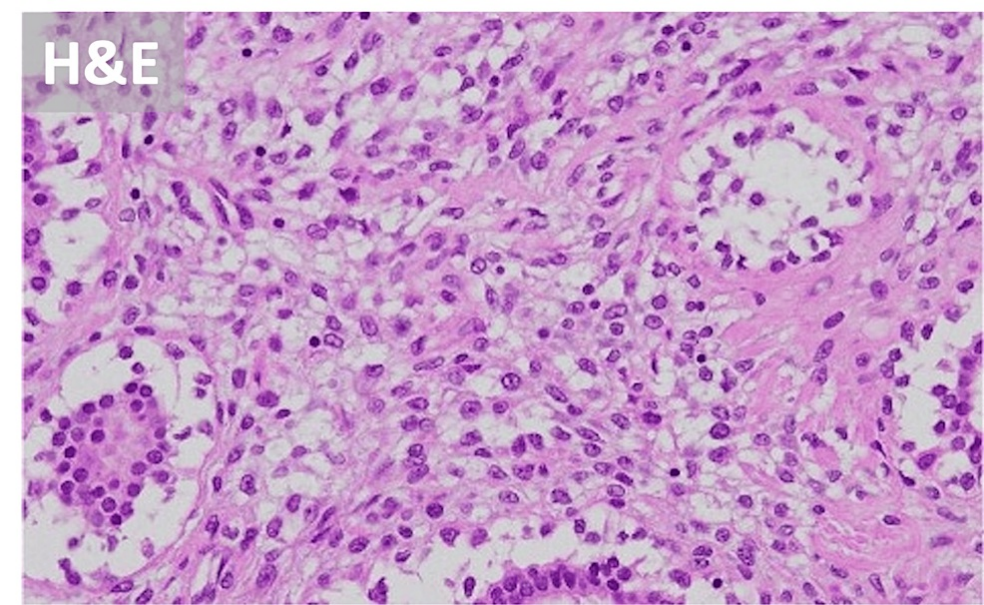
Histopathological examination Histopathological examination revealed a mixture of epithelial and stromal components, and the increased stromal component caused cleft- and leaflike appearance of the glandular ducts, resulting in a lobular morphology.

A needle biopsy was performed to confirm the diagnosis. The patient’s condition was stable, and based on the results of the pathological examination, elective surgery was scheduled. However, three days later, she presented to our emergency department with an increase in erupted mass and active bleeding from the erupting mass (Figure 1B). Upon presentation, her blood pressure was 146/113 mmHg, and her heart rate was 117 beats/min. Active bleeding from the ruptured mass was initially controlled with compression. However, blood tests revealed a Hb level of 11.2 g/dL. She was admitted for immediate follow-up; however, the bleeding persisted throughout the next day, and blood test results demonstrated a further decrease in Hb to 8.9 g/dL. A CT revealed a 105×75 mm mass with contrast effects and a 100×45 mm mass erupting from the paramammary nipple (Figure 3B). Pathological examination of the needle biopsy specimen revealed a lobular morphology with a mixture of epithelial and stromal components, leading to the diagnosis of a phyllodes tumor. Given the persistent bleeding from the mass and decreased Hb levels, emergency surgery was performed to ensure hemostasis. Because of the tumor’s large size, a simple right mastectomy was performed to provide a 10 mm margin around the tumor. Pathological examination confirmed the diagnosis of a phyllodes tumor. A preoperative CT scan confirmed the absence of enlarged axillary lymph nodes; thus, it was determined that axillary lymph node biopsy and dissection were not necessary. As the tumor may have invaded the pectoralis major muscle, a portion of the muscle adjacent to the tumor was excised in concomitant resection. Histopathological examination revealed a mixture of epithelial and stromal components. The increased stromal component caused a cleft- and leaf-like appearance of the glandular ducts, resulting in a lobular morphology, further confirming the diagnosis of phyllodes tumor (Figure 4). The tumor exhibited six mitoses/10 high power fields but was considered malignant because of high stromal cell density and stromal atypia. No evidence of tumor invasion into the pectoralis major muscle was observed.

**Table 2:** Changes in hemoglobin

	First visit	Three days later	Four days later	Postoperative day one	Reference range
Hemoglobin	13.2 g/dL	11.2 g/dL	8.9 g/dL	10.7 g/dL	12-16 g/dL

A blood test performed on the day after surgery revealed an increase in the Hb levels to 10.7 g/dL. The patient recovered and was discharged on postoperative day 10. No additional postoperative radiotherapy was administered. Two years and 10 months have passed since the surgery, and no recurrence or complications have been observed.

## Discussion

Phyllodes tumors are a rare form of breast cancer characterized by good mobility, abundant blood flow within the mass, lack of pain, and rapid growth [1]. The standard treatment is surgical excision of the tumor with a clear margin of healthy tissue (usually 1 cm) around it, which is associated with a low local recurrence rate [6]. However, a simple mastectomy is recommended when the tumor-to-breast ratio is high or when sufficient margins cannot be secured [8]. In this case, owing to the tumor’s large size, a simple right mastectomy was performed to ensure clear margins around the tumor.

Ruptured phyllodes tumors are extremely rare, with only a few reported cases. Herein, we reviewed six reported cases of ruptured phyllodes tumors [2-3,5,8-10] (Table 1). In a case reported by Yang et al. [10], active bleeding from a ruptured phyllodes tumor required emergency surgery to control the bleeding. In this case, the mass rapidly enlarged over a short period (two months) and was thought to have ruptured because of the rate of tumor growth and skin invasion. The cases of ruptured phyllodes tumors were characterized by large masses, ranging from approximately 9 to 21 cm in size. Except for the case reported by Nabi et al. [3], all cases involved borderline or malignant tumors. Nabi et al. [3], who encountered a benign tumor, performed a local excision, whereas all other patients underwent a simple mastectomy. Phyllodes tumors primarily metastasize hematogenously and do not generally require axillary lymph node dissection unless there are enlarged axillary lymph nodes or obvious metastases because of the low frequency of lymph node metastasis. Axillary lymph node metastasis has not been observed in cases of ruptured phyllodes tumors. Despite the substantial size of the breast masses, distant metastases were not observed. Although additional postoperative chemotherapy or radiotherapy is considered on a case-by-case basis in phyllodes tumors, no clear evidence exists [4]. Therefore, no additional postoperative chemotherapy or radiotherapy was administered in this case. Moreover, additional postoperative chemotherapy or radiotherapy has not been reported in the management of other cases of ruptured phyllodes tumors [2-3,5,8-10]. Meher et al. [2] previously reported one case of sudden, unexplained death at home 10 months postoperatively, despite the patient appearing to recover well beforehand. However, the majority of reported patients did not experience recurrence.

**Table 3:** Summaries of previous case reports regarding ruptured phyllodes tumors

Author name, publication year	Age (years)	Grade	Mass size (cm)	Operation	Axillary lymph node metastasis	Distant metastasis	Follow-up after operation
Wijeyaratne, 2010 [9]	48	Borderline	Unknown	Elective, simple mastectomy	No	No	4 years, no recurrence
Nabi et al., 2013 [3]	32	Benign	9.5*8.5*4.5	Elective, wide local excision	No	No	9 months, no recurrence
Ditsatham et al., 2016 [8]	58	Malignant	13.4*14.8*14.3	Elective, simple mastectomy	No	No	4 months, no recurrence
Meher et al., 2016 [2]	60	Malignant	27*20*16	Emergency, simple mastectomy	No	No	10 months, no recurrence, sudden death
Bruce NR, 2017 [5]	60	Malignant	9.2*8.6	Elective, simple mastectomy	No	No	Unknown
Yang M, 2022 [10]	70	Borderline	15*19*21	Emergency, simple mastectomy	No	No	Unknown

## Conclusions

Rapidly growing phyllodes tumors can rupture. Emergency surgery is required in cases of ruptured phyllodes tumors with active bleeding. Although most ruptures of phyllodes tumors occur in borderline or malignant tumors, the incidence of lymph node metastasis, distant metastasis, and postoperative recurrence remains low.

## References

[1] Guerrero MA, Ballard BR, Grau AM (2003). Malignant phyllodes tumor of the breast: review of the literature and case report of stromal overgrowth. Surg Oncol.

[2] Meher S, Mishra TS, Sasmal PK, Rath S, Sharma R (2016). An ulcerated giant malignant phyllodes tumour presenting in septic shock. J Clin Diagn Res.

[3] Nabi J, Akhter SM, Authoy FN (2013). A case of large phyllodes tumor causing "rupture" of the breast: a unique presentation. Case Rep Oncol Med.

[4] Ofri A, Stuart KE, Chan B (2022). Diagnosis and management of phyllodes tumours for the surgeon: an algorithm. Surgeon.

[5] Bruce NR, Carlson JT, Barnard KJ, Henry-Tillman R (2017). Phyllodes tumor masquerading as a fungating breast mass. J Surg Case Rep.

[6] Reinfuss M, Mituś J, Duda K, Stelmach A, Ryś J, Smolak K (1996). The treatment and prognosis of patients with phyllodes tumor of the breast: an analysis of 170 cases. Cancer.

[7] Adesoye T, Neuman HB, Wilke LG, Schumacher JR, Steiman J, Greenberg CC (2016). Current trends in the management of phyllodes tumors of the breast. Ann Surg Oncol.

[8] Ditsatham C, Somwangprasert A, Watcharachan K, Wongmaneerung P (2016). "Ruptured" malignant phyllodes tumor of the breast: a case report. Int Med Case Rep J.

[9] Wijeyaratne SM (2010). Breast 'rupture' due to a phyllodes tumour. BMJ Case Rep.

[10] Yang M, Cui RB, Perera R (2022). Emergency resection of large, ulcerating phyllodes tumour with active bleeding. ANZ J Surg.

